# High prevalence of azole resistance among environmental *Aspergillus fumigatus* isolates from outdoor air in Madrid, Spain

**DOI:** 10.3389/fmicb.2025.1722314

**Published:** 2026-01-23

**Authors:** Juan Carlos Soto-Debrán, Francisco Javier Sánchez-Íñigo, Alejandro B. Calvo-López, Laura Alguacil-Cuéllar, Anastasiia A. Hrynzovska, Emilia Mellado, Saul García Dos Santos, Laura Alcazar-Fuoli, Ana Alastruey-Izquierdo

**Affiliations:** 1 Mycology Reference Laboratory, National Centre for Microbiology, Instituto de Salud Carlos III, Madrid, Spain; 2 Air Quality National Reference Laboratory, National Environmental Health Centre, Instituto de Salud Carlos III, Madrid, Spain; 3 Department of Microbiology and Parasitology with Basics of Immunology of the Bogomolets National Medical University, Kyiv, Ukraine; 4 Center for Biomedical Research in Network in Infectious Diseases (CIBERINFEC-CB21/13/00105), Instituto de Salud Carlos III, Madrid, Spain

**Keywords:** airborne fungi, antifungal resistance, *Aspergillus fumigatus*, environmental fungi, genotyping, One Health

## Abstract

**Introduction:**

*Aspergillus fumigatus* has been designated by the World Health Organization (WHO) as a critical fungal pathogen. Its spores are commonly present in the air and are inhaled daily. Azoles are the first-line treatment for *Aspergillus* infections, but the emergence of resistance is a growing concern. However, limited data exist on the occurrence of azole-resistant *A. fumigatus* in the outdoor environment in Spain.

**Methods:**

This study aimed to investigate the prevalence of azole-resistant *A. fumigatus* isolates in outdoor air at two distinct locations in Madrid. We characterized the isolates using TRESPERG genotyping and examined the underlying molecular mechanisms responsible for azole resistance development.

**Results:**

Azole-resistant *A. fumigatus* isolates were found in 55% of the 20 air samples collected. Among the 200 *A. fumigatus* isolates analyzed, 38.5% were azole resistant and were classified into 10 different genotypes. Notably, the TR34/L98H mutation in Cyp51A was found in 77% of the resistant isolates, while 23% showed no mutations in the screened targets (*cyp*51A, *cyp*51B, or *hmg*1).

**Discussion:**

This study revealed a high prevalence of azole-resistant *A. fumigatus* in outdoor environmental air, with the TR34/L98H mutation being the main mechanism of azole resistance. A close genetic relationship was observed among the resistant isolates. This research underscores the need for continued monitoring of environmental azole-resistant *A. fumigatus* isolates and highlights the importance of understanding genetic diversity and resistance mechanisms to develop effective strategies for fungal infection control.

## Introduction

Fungal infections pose a significant global health burden, affecting over one billion individuals and contributing to an estimated 3.8 million deaths annually, of which 2.5 million are directly attributable to invasive fungal infections (Bongomin et al., 2017; Brown et al., 2012; Seagle et al., 2021; Denning, 2024). Notably, the incidence of fungal infections has exhibited a dynamic pattern in recent years. While advances in prevention and treatment have led to a decrease in various fungal infections among HIV + patients (Rayens et al., 2022), a concurrent increase has been observed among non-HIV immunocompromised individuals. These include patients undergoing chemotherapy (Sipsas and Kontoyiannis, 2012), long-term steroid treatments, allogeneic hematopoietic stem cell transplantation, or solid organ transplantation (Pappas et al., 2010), as well as individuals with diabetes, influenza, or COVID-19 (Feys et al., 2024).

Among molds, *Aspergillus fumigatus* stands out as the main etiologic agent responsible for diverse forms of aspergillosis and has been classified by the World Health Organization (WHO) as one of the four critical pathogens in the first WHO fungal priority pathogens list, due in part to its increasing rates of resistance (WHO, 2022).

Every day, we inhale *A. fumigatus* spores, with the lungs serving as the main point of entry for all forms of aspergillosis (Segal, 2009). The increase in airborne concentrations of *A. fumigatus* conidia represents a risk factor for aspergillosis infection (O’Gorman, 2011). Furthermore, the release and airborne dispersion of conidia from reservoirs such as compost and accumulated dust have also been associated with various diseases, including allergic bronchopulmonary aspergillosis (ABPA) and hypersensitivity pneumonitis in workers at landfills (O’Gorman, 2011).

Azoles are the frontline treatment for *Aspergillus* infections, but the emergence of resistance is an alarming concern. Azole-resistant *A. fumigatus* strains have been documented on every continent, with Europe reporting the highest clinical rates (Song et al., 2025). A combination of mutations in the *cyp51A* gene, such as TR_34_/L98H and TR_46_/Y121F/T289A, has been reported as the dominant mechanism of azole resistance in *A. fumigatus,* and their development has been linked to the use of azole fungicides in agriculture (Mellado et al., 2007).

Investigations conducted in various parts of the world, examining soil, dust, and indoor and outdoor air to detect the presence of azole-resistant *A. fumigatus*, have reported a wide range of resistance rates depending on the sample origin and type, ranging from isolated cases to rates exceeding 30% (Zahra Vaghar et al., 2022; Gonçalves et al., 2021; Ener et al., 2022; Sewell et al., 2019; Shelton et al., 2022; Tsitsopoulou et al., 2018; Resendiz-Sharpe et al., 2021; Wang et al., 2018; Viegas et al., 2022; Van Der Linden et al., 2013; Sabino et al., 2021; Paluch et al., 2019; Toyotome et al., 2017; Onishi et al., 2017; Gonzalez-Jimenez et al., 2021; Godeau et al., 2020; Dunne et al., 2017; Chowdhary et al., 2012; Chen et al., 2019; Bromley et al., 2014; Ahmad et al., 2014; Alvarez-Moreno et al., 2019; Alvarez-Moreno et al., 2017; Lucio et al., 2022; Snelders et al., 2009; Mortensen et al., 2010; Monteiro et al., 2019; Duong et al., 2021; Arendrup et al., 2024). The prevalence of azole resistance in Spain has been primarily studied in *A. fumigatus* isolates from clinical samples, with rates increasing from below 1% in 2010–11 (Alastruey-Izquierdo et al., 2013) and 1.2% in 2016 (Alastruey-Izquierdo et al., 2018) to 5.5% in the most recent study in 2021 (Escribano et al., 2021). Higher azole resistance rates have been documented in several other European countries, highlighting notable geographic variability in resistance prevalence (Song et al., 2025). While these studies indicate an alarming rise in resistance in clinical settings, scant data exist regarding the presence of azole-resistant *A. fumigatus* isolates in the outdoor environment in Spain.

The primary objective of this study was to assess the prevalence of azole-resistant *A. fumigatus* isolates in the outdoor air at two distinct locations in Madrid. In addition, we aimed to conduct genotyping analyses and characterize the underlying molecular mechanisms of azole resistance.

## Materials and methods

### Sampling and identification of *Aspergillus fumigatus* isolates in ambient air samples

A total of 20 individual air samples were obtained during 10 sampling campaigns carried out between July 2021 and June 2022 simultaneously at two different locations in Madrid, Spain: Semiurban A (lower population density and predominance of residential and green areas; coordinates: 40°27′28.2”N 3°51′49.5”W) and urban B (situated within a densely populated area with predominantly built-up surroundings; coordinates: 40°20′33.3”N 3°42′45.1”W). The samples were obtained by filtering ambient air in accordance with the CEN (European Committee for Standardization) Technical Specification (TS) UNE CEN/TS 16115-1:2013 (CEN/TS 16115-1:2013, 2013). For each sample, a total of 12 m^3^ of ambient air was filtered. Total suspended particles (TSP) were collected using a vacuum pump with a calibrated flow (ML-80 header attached to a LVS 3.1/MVS 6.1 pump; COMDE-DERENDA, Germany) over 4 h through 3 μm pore-size sterilized gelatin/polycarbonate filters (Sartorius, Germany) using a Low/Medium Volume reference sampler (LVS 3.1/MVS 6.1 models, COMDE-DERENDA) equipped with a specific inlet for this purpose (ML-80 model, COMDE-DERENDA). The samplers were calibrated with an annual frequency in terms of flow rate, ambient temperature, and ambient pressure. Each sampling lasted 4 h and was carried out at a flow rate of 50 L/min (3 m^3^/h). The duration and flow rate for the samplings in this study were determined through a validation process (data not shown), which involved several field campaigns performed at the ISCIII-Majadahonda air quality monitoring station using the same samplers, inlets, and filters. Various sampling durations and flow rates were tested until optimal results in terms of fungal growth were obtained.

Subsequently, following UNE CEN/TS 16115-1:2013, the exposed filters were dissolved in a saline solution (0.85% NaCl) with 0.01% Tween 80, and immediately after, direct suspensions and 10- and 100-fold dilutions were cultured on Sabouraud agar plates (Sigma-Aldrich, Spain). The plates were incubated at 30 °C for 2 days and frequently observed to subculture all growing fungal colonies onto potato-dextrose agar tubes (Oxoid, Madrid, Spain). The total number of colony-forming units (CFUs) per plate was recorded. All CFUs from each plate were subcultured and initially assessed based on macroscopic morphology. Subsequently, they were identified based on their ribosomal proteins using MALDI-TOF with the commercial VITEK^®^ MS Mould Kit for protein extraction and analyzed against the VITEK^®^ MS system v3.2 IVD and Saramis v4.16 RUO database (Zvezdánova et al., 2022; McMullen et al., 2016) (Biomerieux, Spain).

Fungal DNA extraction was performed to confirm *A. fumigatus sensu stricto* isolates and to classify all isolates that were not identified by MALDI-TOF MS. For DNA extraction, the isolates were subcultured in glucose–yeast extract–peptone (GYEP) liquid medium (0.3% yeast extract and 1% peptone; Difco, Spain) with 2% glucose (Sigma-Aldrich, Spain) for 24 h-48 h at 30 °C. After mechanical disruption of the mycelium by vortex mixing with glass beads, genomic DNA of the isolates was extracted using the phenol-chloroform method (Tang et al., 1992). Subsequently, molecular identification was performed by PCR amplification and sequencing of a portion of the *β*-tubulin gene (Balajee et al., 2005) under conditions previously described (Alastruey-Izquierdo et al., 2013). Sequences were edited and analyzed using the DNAStar Lasergene 12 software (DNAStar Inc., USA) and compared with reference sequences from the GenBank,^1^ as well as an in-house database constructed in Bionumerics 8.1 (Biomerieux, Spain), which contains over 17,000 sequences from isolates and reference sequences representing more than 350 different fungal species.

### Antifungal susceptibility testing

Antifungal susceptibility screening was performed on all confirmed *A. fumigatus* isolates following the European Committee on Antifungal Susceptibility Testing (EUCAST) reference method 10.1 (Guinea et al., 2019), as previously described (Lucio et al., 2022), using the following concentrations: 4 mg/L itraconazole, 2 mg/L voriconazole, and 0.5 mg/L posaconazole (Sigma-Aldrich, Spain). Plates were incubated at 35 °C for 48 h, and isolates were considered non-susceptible when growth was observed.

Non-susceptible isolates were confirmed following the EUCAST microdilution reference method 9.4 (Guinea et al., 2022). Antifungal susceptibility testing was performed using the following antifungals at the given concentration ranges: amphotericin B (0.03 to 16 mg/L, Sigma-Aldrich Química, Madrid, Spain), itraconazole (0.016 to 8 mg/L, Sigma-Aldrich Química, Madrid, Spain), voriconazole (0.016 to 8 mg/L, Sigma-Aldrich Química, Madrid, Spain), posaconazole (0.016 to 8 mg/L, Sigma-Aldrich Química, Madrid, Spain) and isavuconazole (0.016 to 8 mg/L; Sigma-Aldrich Química, Madrid, Spain).

*Aspergillus flavus* ATCC 204304 and *A. fumigatus* ATCC 204305 were used as quality control strains in all tests. Plates were incubated at 35 °C, and minimum inhibitory concentrations (MICs) for amphotericin B, itraconazole, voriconazole, posaconazole, and isavuconazole were determined after 48 h. The EUCAST clinical breakpoints were used to define resistance (EUCAST, 2020).

### Genotyping and characterization of molecular mechanisms of azole resistance

All isolates that were resistant to at least one azole were genotyped using the TRESPERG typing method described previously (Garcia-Rubio et al., 2016; Garcia-Rubio et al., 2018). One randomly selected azole-susceptible *A. fumigatus* isolate from each plate containing resistant isolates was also typed. Relationships between genotypes were visualized by constructing a minimum spanning tree using Bionumerics 8.1 (Biomerieux, France), treating the data as categorical information.

Molecular mechanisms of azole resistance were investigated in all isolates that were resistant to at least one azole by amplifying and sequencing the full *cyp51*A gene, including its promoter, as previously described (Alastruey-Izquierdo et al., 2013). *Aspergillus fumigatus* strain A1163 (NCBI accession number DS499598.1) was used as a reference. *Cyp*51B (Gonzalez-Jimenez et al., 2020) and *hmg1* (Gonzalez-Jimenez et al., 2021) mutations were also investigated in resistant isolates without *cyp*51A mutations, as previously described.

## Results

### Identification of *Aspergillus fumigatus* in air samples and antifungal susceptibility testing

A total of 20 ambient-air TSP samples were analyzed across 10 different time points at two different locations in Madrid. *A. fumigatus* was isolated in 19 of the 20 (95%) air samples. A total number of 200 *A. fumigatus* CFUs were obtained, representing the most frequent species at 42% of the total 477 isolates. Azole-resistant *A. fumigatus* isolates were found in 11 (55%) of the 20 air samples (Table 1).

**Table 1:** Number of *A. fumigatus* CFUs and azole-resistant *A. fumigatus* CFUs per sampling location.

Location	No. of air samples			No. of CFUs		
	Total	With *A. fumigatus* (% over total)	With resistant *A. fumigatus* (% over total)	Total	*A. fumigatus* (% over total CFUs)	Resistant *A. fumigatus* (% of *A. fumigatus* CFUs)
Semiurban	10	10 (100%)	6 (60%)	222	109 (49%)	28 (26%)
Urban	10	9 (90%)	5 (50%)	255	91 (36%)	49 (54%)
Total	20	19 (95%)	11 (55%)	477	200 (42%)	77 (39%)

**Figure 1:**
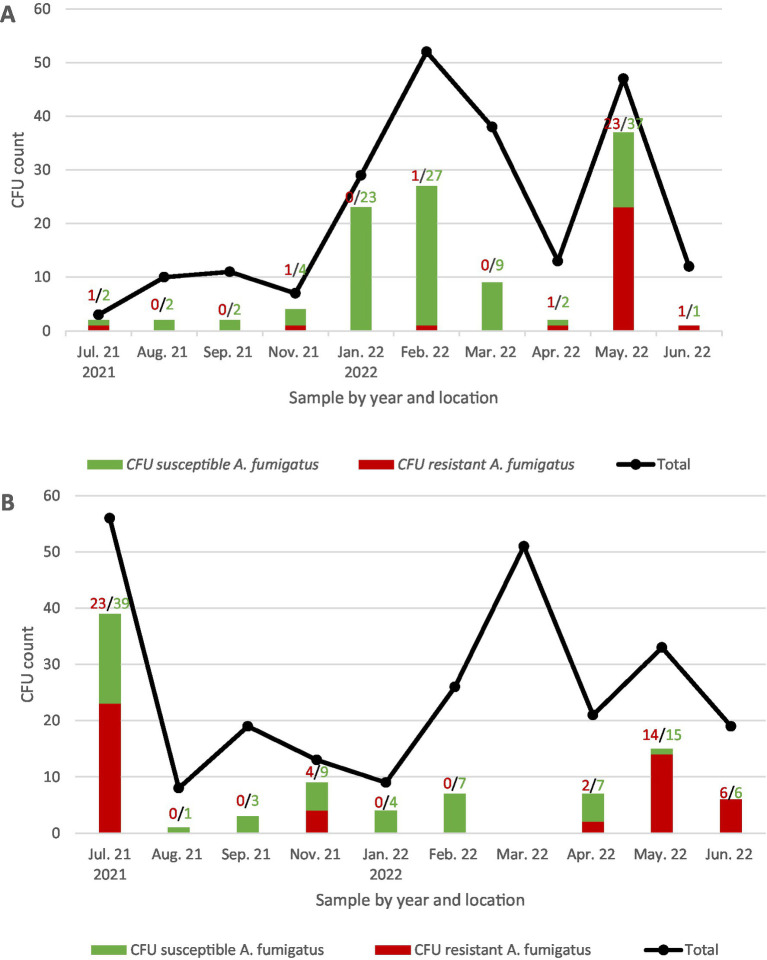
Temporal distribution of CFUs of susceptible and resistant *Aspergillus fumigatus* isolates from July 2021 to June 2022 at two locations (semiurban **A** and urban **B**). The numbers above the columns indicate the number of resistant isolates (red) over the total *A. fumigatus* isolates (green).

The temporal distribution and azole-resistance profile of *A. fumigatus* CFUs per sampling event and location are shown in Figure 1.

Of the *A. fumigatus* isolates, 38.5% (*n* = 77) were classified as non-susceptible in the screening to at least one azole. As shown in Figure 1, the percentage of non-susceptible isolates compared to the total CFU varied among the samples, ranging from 4 to 100%.

The EUCAST microdilution method confirmed that 38.5% (*n* = 77) of the *A. fumigatus* isolates were azole-resistant. Among these, 37.5% (*n* = 75/77) were resistant to isavuconazole, 36% (72/77) to posaconazole, 34.5% (69/77) to itraconazole, and 26% (52/77) to voriconazole. In addition, 1.5% (3/77) of isolates were classified as resistant to amphotericin B. MICs of all azole-resistant isolates are provided in Supplementary Table S1.

### Genotyping and characterization of molecular mechanisms of azole resistance

**Figure 2:**
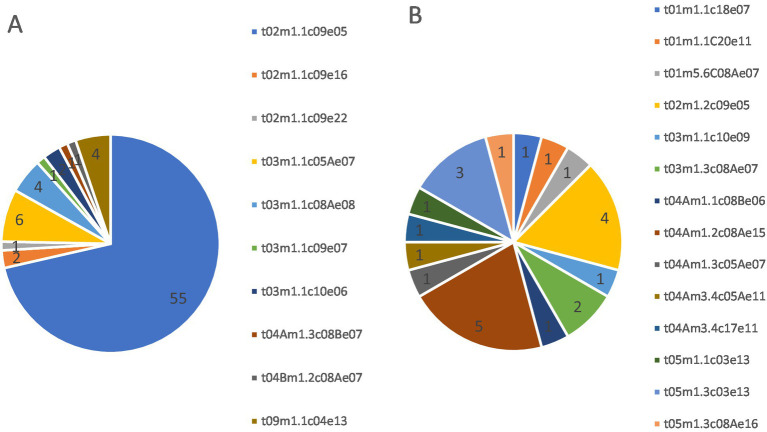
Tresperg typing distribution of **(A)** azole-resistant (*n* = 77) and **(B)** selected azole-susceptible (*n* = 24) *A. fumigatus* isolates. One susceptible isolate per plate from which a resistant isolate was recovered was randomly selected for typing.

TRESPERG analysis revealed that the 77 resistant isolates were classified into 10 different genotypes, while the 24 susceptible isolates belonged to 14 unique genotypes. We did not detect any overlap between genotypes among the susceptible or resistant isolates (Figure 2).

Among the resistant isolates, 55 (71.4%) were classified as type t02m1.1c09e05. This genotype was found in eight different samples across both locations. The remaining resistant isolate types were unique to each sample, except for type t03m1.1c08Ae08, which was found in two samples from the same location (February and May 2022 in a semiurban location). Types t02m1.2c09e05 and t04Am1.2c08Ae15 were found in the same sampling campaign at both locations, and types t03m1.3c08Ae07 and t05m1.3c03e13 were found in different campaigns within the same location.

**Figure 3:**
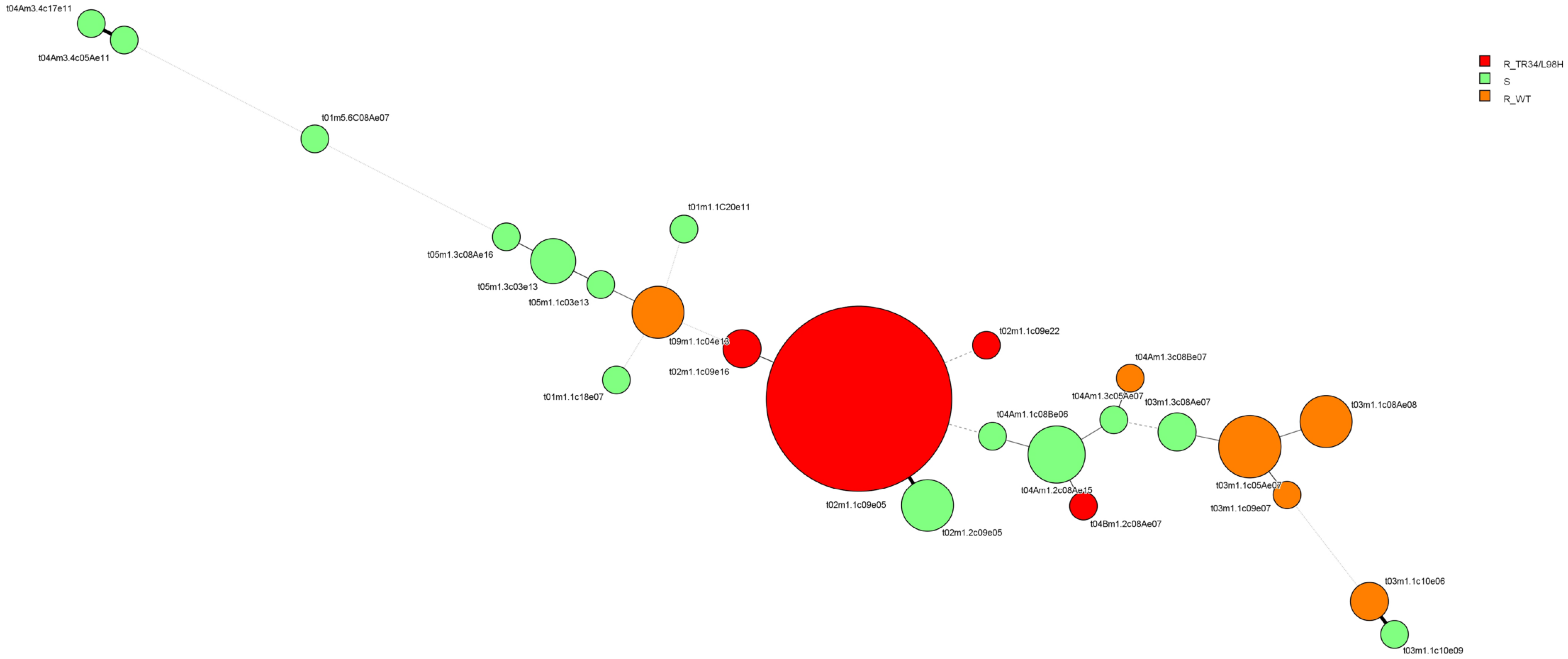
Minimum spanning tree showing the genetic diversity of susceptible and azole-resistant *A. fumigatus* isolates. Each circle represents a TRESPERG genotype. The circle size is related to the number of isolates with the same genotype. Straight bold lines denote groups that differ by only one marker. Solid lines indicate differences in two markers, and dashed lines indicate differences in three or more markers. Red: resistant isolates with the TR_34_/L98H *cyp51A* mutation. Orange: resistant isolates with WT *cyp51A*; green: susceptible isolates. For more information on the isolates in each genotype, see Supplementary Table S1.

Regarding resistance mechanisms, the TR_34_/L98H mutation in Cyp51A was found in 59 (77%) of the resistant isolates, while 18 (23%) of the isolates showed no mutations in any of the screened targets (*cyp51A, cyp51B,* or *hmg1*). A total of 45 of the 59 (93.2%) isolates carrying the TR_34_/L98H mutation in Cyp51A belonged to the most frequent genotype (t02m1.1c09e05). Another three isolates with this resistance mechanism belonged to two different but closely related genotypes (two isolates to t02m1.1c09e16 and one to t02m1.1c09e22), and the remaining TR_34_/L98H isolate belonged to a more distant genotype, t04Bm1.2c08Ae07. Resistant isolates with wild-type *cyp51A* were grouped into six different genotypes (Figure 3).

## Discussion

*Aspergillus fumigatus* is the most common mold causing human infections, primarily acquired through the inhalation of airborne conidia. While the prevalence of azole-resistant *A. fumigatus* in Spain is on the rise in clinical settings, there is a significant gap in studies evaluating its prevalence in the environment (Alastruey-Izquierdo et al., 2013; Alastruey-Izquierdo et al., 2018; Escribano et al., 2021).

Several studies around the globe have detected azole-resistant *A. fumigatus* in diverse environments, including urban, rural, agricultural, and hospital settings (Zahra Vaghar et al., 2022; Gonçalves et al., 2021; Ener et al., 2022; Sewell et al., 2019; Shelton et al., 2022; Tsitsopoulou et al., 2018; Resendiz-Sharpe et al., 2021; Wang et al., 2018; Viegas et al., 2022; Van Der Linden et al., 2013; Sabino et al., 2021; Paluch et al., 2019; Toyotome et al., 2017; Onishi et al., 2017; Gonzalez-Jimenez et al., 2021; Godeau et al., 2020; Dunne et al., 2017; Chowdhary et al., 2012; Chen et al., 2019; Bromley et al., 2014; Ahmad et al., 2014; Alvarez-Moreno et al., 2019; Alvarez-Moreno et al., 2017; Lucio et al., 2022; Snelders et al., 2009; Mortensen et al., 2010; Monteiro et al., 2019). The majority of the studies published to date focus on two main types of environments. On the one hand, compost and soil samples, which tend to harbor a higher fungal load, are considered reservoirs and potential hotspots for antifungal resistance development (Song et al., 2025). On the other hand, indoor air from clinical settings, households, and workspaces where particular aspergillosis cases can be detected is being assessed to contribute to better indoor management (O’Gorman, 2011).

In Spain, few studies have focused on the prevalence of fungi in environmental air samples (Guinea et al., 2006; Elvira-Rendueles et al., 2013; Vélez-Pereira et al., 2019; Antón et al., 2019). Regarding azole-resistant airborne *A. fumigatus* in Spain, most of the limited data reported to date focus on monitoring indoor air in domestic environments and hospitals (Gonzalez-Jimenez et al., 2021; Peláez-García de la Rasilla et al., 2022; Guinea et al., 2005). Notably, in the largest Spanish study published in 2005, which included outdoor air, no azole-resistant isolates were found among the 310 environmental samples analyzed (175 from outdoor air and 135 from hospital air) (Guinea et al., 2005). In contrast, our study revealed a substantial rate of azole-resistant isolates (38.5%). A previous study in occupational environments in Portugal led by Gonçalves et al. reported a 3% rate of azole-resistant *A. fumigatus,* with one isolate identified in indoor air (Gonçalves et al., 2021). In addition, Monteiro et al. (2019) reported a 21.8% rate of azole-resistant *A. fumigatus* in environmental air collected from hospitals and water-sorting plants. However, differences in methodology among these studies, such as sample collection methods and incubating conditions, make direct data comparison complex. For instance, while Guinea et al. collected 200 L of air by impaction onto Sabouraud dextrose and Czapek agar plates, Gonçalves collected different kinds of samples, from 50 to 250 L of air via impaction to settled dust, surface swabs, and pieces of personal protection devices, all cultured at 27 °C for 5 to 7 days. Finally, Monteiro collected 50–100 L of air by impaction on agar plates incubated at 25 °C, as well as 500–1,000 L of air incubated at 45 °C. In our study, we partnered with the National Centre for Environmental Health and employed an automated sampling method using an ML-80 header attached to a LVS 3.1/MVS 6.1 pump (COMDE-DERENDA, Germany) that filters ambient air in accordance with UNE CEN/TS 16115-1:2013 recommendations (CEN/TS 16115-1:2013, 2013). While all these procedures provide valuable information regarding azole resistance in environmental samples, there is a clear need for a standardized surveillance protocol with harmonized data acquisition to facilitate meaningful data comparison.

Sampling was performed during the daytime, and basic meteorological parameters (ambient temperature, relative humidity, and air pressure) were recorded during each campaign. However, the limited number of sampling points and campaigns and the monthly sampling frequency did not allow for a meaningful analysis of potential associations between these variables and the abundance or azole resistance rates of *A. fumigatus*. Future studies with higher temporal resolution will be required to determine whether environmental conditions influence fungal load or resistance patterns. Only three isolates showed amphotericin B MICs of 2 mg/L, a single dilution above the EUCAST resistance breakpoint (1 mg/L). Given the rarity of amphotericin B resistance in *A. fumigatus* and the known variability in amphotericin B MIC measurements, these borderline elevations likely reflect natural variability rather than a stable resistance phenotype. However, this should be further investigated, as some studies have reported increased rates of amphotericin B resistance among clinical (Fakhim et al., 2022) and environmental (Ashu et al., 2018) isolates of *A. fumigatus*.

Fast and reliable methods for screening azole-resistant *A. fumigatus* isolates are available (Lucio et al., 2022). The combination of air sampling, identification, and azole resistance screening has resulted in the development of a robust and reliable methodology that can be adapted and incorporated into surveillance networks.

The TR_34_/L98H mutation in Cyp51A is the most frequent azole resistance mechanism in *A. fumigatus* and has been detected globally (Garcia-Rubio et al., 2017). It has been associated with resistance development in the environment (Snelders et al., 2009), but it is also found in clinical settings (Rhodes et al., 2022). In this study, 77% of the azole-resistant isolates carried this mutation, with no other resistance mechanisms found among the examined genes. Other environmental studies from countries including China, Colombia, Denmark, France, India, Italy, Iran, Kuwait, the UK, and the USA have also found TR_34_/L98H to be the dominant phenotype in various samples, such as air, dust, flower bulbs, and soils from clinical settings (Paluch et al., 2019; Godeau et al., 2020; Chowdhary et al., 2012; Ahmad et al., 2014; Mortensen et al., 2010), as well as in crop soils, composts, and waste (Chowdhary et al., 2012; Bromley et al., 2014; Alvarez-Moreno et al., 2019; Arendrup et al., 2024; Hurst et al., 2017; Chen et al., 2016; Trovato et al., 2018; Vaezi et al., 2018; Rocchi et al., 2018; Chen et al., 2020). No TR_46_/Y121F/T289A mutations were identified in our dataset, which is consistent with previous Spanish studies reporting TR_46_ as a rare mechanism of resistance in both clinical and environmental isolates (Alastruey-Izquierdo et al., 2013; Alastruey-Izquierdo et al., 2018; Escribano et al., 2021).

We found that 23% of the resistant isolates had unknown mechanisms of resistance. Other environmental studies worldwide have reported azole-resistant strains with a wild-type *cyp51A*, with prevalence rates ranging from 4.7 to 83.3% (Tsitsopoulou et al., 2018; Wang et al., 2018; Bromley et al., 2014; Alvarez-Moreno et al., 2017; Snelders et al., 2009; Monteiro et al., 2019; Vaezi et al., 2018; Chen et al., 2020; Cao et al., 2020). Recent extensive surveillance studies of clinical isolates have also reported that 20% of azole-resistant isolates carry a wild-type *cyp51A* (Risum et al., 2022; Khojasteh et al., 2023). Although several non-*cyp51A* resistance mechanisms have been described (Nywening et al., 2020; Sharma et al., 2019), their prevalence remains relatively low. In our investigation, we were unable to find mutations in either *cyp51B* or *hmg1* among the azole-resistant isolates with WT *cyp51A*. A recent study reported that up to 78% of azole-resistant clinical isolates had no mutations in either *cyp51A* or *hmg1* (Resendiz-Sharpe et al., 2020).

The TRESPERG method is a useful tool for performing genotyping studies due to its specificity and discriminatory power (Garcia-Rubio et al., 2018). It has been compared with the STR*Af* (De Valk et al., 2005) method, yielding comparable results (Garcia-Rubio et al., 2018). The TRESPERG method does not require trained personnel, specialized equipment, or software for analysis. Nevertheless, the lack of publicly maintained databases for results comparisons across studies complicates data interpretation, especially when identifying new types. Although the use of this method is not yet widespread, our study identified genotypes common to those described by Gonzalez-Jimenez et al. Notably, some environmental *A. fumigatus* strains carrying the TR_34_/L98H mutation, isolated from the bathroom air of a room occupied by an *Aspergillus-*colonized patient (Gonzalez-Jimenez et al., 2021), shared the same genotype as some of our TR_34_/L98H isolates (t02m1.1c09e16). In addition, several genotypes identified in our study (in both resistant and susceptible isolates) are closely related to genotypes previously isolated from patients and associated environments in published studies (Gonzalez-Jimenez et al., 2021; Peláez-García de la Rasilla et al., 2022).

Azole-resistant isolates carrying the TR_34_/L98H mutation in this study were typed as t02 and t04B CSP types (55 isolates as t02m1.1c09e05, two as t02m1.1c09e16, one as t02m1.1c09e22, and one as t04Bm1.2c08Ae07), aligning with other European isolates (Gonzalez-Jimenez et al., 2021; Camps et al., 2012; Bader et al., 2015; Fraaije et al., 2020; Bader, 2021) and indicating a close genetic relationship and a common origin for this resistance mechanism. Azole-resistant *A. fumigatus* isolates with wild-type Cyp51A in this study were mainly classified as the t03 CSP type (six isolates as t03m1.1c05Ae07, four as t03m1.1c08Ae08, one as t03m1.1c09e07, and two as t03m1.1c10e06). Similar genotypes have been reported in other studies (Bader et al., 2015; Steinmann et al., 2014; van der Torre et al., 2021; Bader et al., 2013), suggesting a common genetic background.

Studies of genotypic diversity in *A. fumigatus* have shown higher diversity among susceptible isolates and greater genetic relatedness among resistant ones (Garcia-Rubio et al., 2018), in both clinical and environmental isolates (van der Torre et al., 2021; Jeanvoine et al., 2020; Ahangarkani et al., 2020; Rocchi et al., 2021). Our findings were consistent with this pattern, as we classified 78 resistant isolates into 11 different genotypes, while the 34 susceptible isolates were grouped into 20 genetic types.

Despite the contributions of our study, several limitations should be acknowledged. First, regarding the representativeness of the sampling locations, the sites were chosen to cover both semiurban and urban settings. In both cases, the sites met the macroscale and microscale siting criteria established by the European Directive 2008/50/CE [European Union (EU), 2008] and can therefore be considered representative of the larger surrounding area. However, because sampling was restricted to two locations within the city of Madrid and was conducted only once per month, the findings cannot be considered representative of the entire region or of broader national environmental patterns. Second, the selection of the sampling flow rate and duration was based on a standardization process (data not shown) to optimize the recovery of CFUs. Third, the choice of sampling inlets and filters is crucial to effectively capture environmental fungi while minimizing fungal loss during sampling. All these parameters were standardized following the CEN/TS 16115-1:2013 (2013) Technical Specification and the manufacturer’s instructions. Lastly, our study focused on cultivable molds capable of growing under specific conditions of time, temperature, and medium. Therefore, our results reflect the fungi circulating in the air during the specific days and times under the selected conditions, but they cannot be extrapolated to represent the fungal population circulating in the air throughout the entire month. Nevertheless, the results are impactful due to the consistent detection of resistant isolates in the sampling conditions and along the months monitored.

In conclusion, our study reported a high prevalence of azole-resistant *A. fumigatus* in outdoor environmental air, with the TR_34_/L98H mutation being the dominant mechanism of azole resistance and a close genetic relationship among the resistant isolates. However, a subset of resistant isolates with unknown mechanisms of resistance underscores the need for further investigations into new mechanisms of antifungal resistance. In addition, there is a pressing need to develop a standardized and harmonized protocol for resistance surveillance within the One Health framework, suitable for different settings, to ensure the comparability of data. Future studies integrating both environmental and clinical isolates from the same geographic area will be important to explore potential links between environmental resistance reservoirs and clinical disease. The implications of azole-resistant *A. fumigatus* in environmental air and its potential correlation with clinical isolates need to be investigated.

## Data Availability

The raw data supporting the conclusions of this article will be made available by the authors, without undue reservation.

## Footnotes

1
http://www.ncbi.nlm.nih.gov/GenBank/
