# Supplementary material for: High prevalence of azole resistance among environmental Aspergillus fumigatus isolates from outdoor air in Madrid, Spain
Source: Front Microbiol. 2026 Jan 23;16:1722314. doi: 10.3389/fmicb.2025.1722314 (PMC12884171; doi:10.3389/fmicb.2025.1722314)
Supplement: Supplementary file 1 [file Table_1.docx]

# Supplementary Data

Table s1. Azole susceptibility profiles, genotype and sample origin of all genotyped strains.

| SAMPLE | DATE | ID | Screening | MICs (mg/L) | | | | *cyp51A* mutations | TRESPERG |
| --- | --- | --- | --- | --- | --- | --- | --- | --- | --- |
|  |  |  |  | IZ | VZ | PZ | ISV |  |  |
| A12 | 28/02/2022 | 307 | S | ND | | | | | t01m1.1c18e07 |
| B2 | 03/08/2021 | 187 | S | ND | | | | | t01m1.1C20e11 |
| B1 | 19/07/2021 | 139 | S | ND | | | | | t01m5.6C08Ae07 |
| B1 | 19/07/2021 | 147 | NS | **16** | **4** | **0.50** | **8** | TR34/L98H | t02m1.1c09e05 |
| B1 | 19/07/2021 | 138 | NS | **16** | **16** | **1** | **16** | TR34/L98H | t02m1.1c09e05 |
| B1 | 19/07/2021 | 156 | NS | **16** | **16** | **0.50** | **8** | TR34/L98H | t02m1.1c09e05 |
| B1 | 19/07/2021 | 150 | NS | **16** | **16** | **2** | **8** | TR34/L98H | t02m1.1c09e05 |
| B1 | 19/07/2021 | 143 | NS | **16** | **16** | **2** | **16** | TR34/L98H | t02m1.1c09e05 |
| B1 | 19/07/2021 | 148 | NS | **16** | **16** | **2** | **16** | TR34/L98H | t02m1.1c09e05 |
| B1 | 19/07/2021 | 161 | NS | **16** | **16** | **1** | **16** | TR34/L98H | t02m1.1c09e05 |
| B1 | 19/07/2021 | 152 | NS | **16** | **8** | **0.50** | **16** | TR34/L98H | t02m1.1c09e05 |
| B1 | 19/07/2021 | 151 | NS | **16** | **8** | **1** | **16** | TR34/L98H | t02m1.1c09e05 |
| B1 | 19/07/2021 | 170 | NS | **16** | **8** | **0.50** | **8** | TR34/L98H | t02m1.1c09e05 |
| B1 | 19/07/2021 | 141 | NS | **16** | **4** | **2** | **16** | TR34/L98H | t02m1.1c09e05 |
| B1 | 19/07/2021 | 145 | NS | **16** | **4** | **0.50** | **8** | TR34/L98H | t02m1.1c09e05 |
| B1 | 19/07/2021 | 154 | NS | **16** | **4** | **0.50** | **8** | TR34/L98H | t02m1.1c09e05 |
| B1 | 19/07/2021 | 164 | NS | **16** | **4** | **0.50** | **8** | TR34/L98H | t02m1.1c09e05 |
| A15 | 17/05/2022 | 519 | NS | **16** | **4** | **2** | **8** | TR34/L98H | t02m1.1c09e05 |
| B1 | 19/07/2021 | 160 | NS | **16** | **4** | **0.50** | **8** | TR34/L98H | t02m1.1c09e05 |
| A15 | 17/05/2022 | 520 | NS | **16** | **4** | **2** | **8** | TR34/L98H | t02m1.1c09e05 |
| B9 | 17/05/2022 | 528 | NS | **16** | **4** | **2** | **8** | TR34/L98H | t02m1.1c09e05 |
| B9 | 17/05/2022 | 534 | NS | **16** | **4** | **2** | **8** | TR34/L98H | t02m1.1c09e05 |
| B1 | 19/07/2021 | 144 | NS | **16** | **4** | **0.50** | **8** | TR34/L98H | t02m1.1c09e05 |
| B1 | 19/07/2021 | 158 | NS | **16** | **4** | **0.50** | **8** | TR34/L98H | t02m1.1c09e05 |
| B10 | 13/06/2022 | 582 | NS | **16** | **16** | **2** | **16** | TR34/L98H | t02m1.1c09e05 |
| B10 | 13/06/2022 | 586 | NS | **16** | **16** | **2** | **16** | TR34/L98H | t02m1.1c09e05 |
| A15 | 17/05/2022 | 514 | NS | **16** | **8** | **1** | **16** | TR34/L98H | t02m1.1c09e05 |
| B10 | 13/06/2022 | 587 | NS | **16** | **8** | **16** | **16** | TR34/L98H | t02m1.1c09e05 |
| B10 | 13/06/2022 | 588 | NS | **16** | **8** | **4** | **16** | TR34/L98H | t02m1.1c09e05 |
| B9 | 17/05/2022 | 551 | NS | **16** | **4** | **2** | **16** | TR34/L98H | t02m1.1c09e05 |
| B9 | 17/05/2022 | 552 | NS | **16** | **4** | **2** | **16** | TR34/L98H | t02m1.1c09e05 |
| A15 | 17/05/2022 | 485 | NS | **16** | **4** | **1** | **8** | TR34/L98H | T02m1.1c09e05 |
| A15 | 17/05/2022 | 488 | NS | **16** | **4** | **1** | **8** | TR34/L98H | t02m1.1c09e05 |
| A15 | 17/05/2022 | 493 | NS | **16** | **4** | **1** | **8** | TR34/L98H | T02m1.1c09e05 |
| A15 | 17/05/2022 | 511 | NS | **16** | **4** | **1** | **16** | TR34/L98H | t02m1.1c09e05 |
| B9 | 17/05/2022 | 548 | NS | **16** | **4** | **2** | **16** | TR34/L98H | t02m1.1c09e05 |
| B10 | 13/06/2022 | 574 | NS | **16** | **4** | **4** | **16** | TR34/L98H | t02m1.1c09e05 |
| B9 | 17/05/2022 | 546 | NS | **16** | **4** | **2** | **16** | TR34/L98H | t02m1.1c09e05 |
| B9 | 17/05/2022 | 553 | NS | **16** | **4** | **2** | **16** | TR34/L98H | t02m1.1c09e05 |
| A16 | 13/06/2022 | 565 | NS | **16** | **4** | **8** | **16** | TR34/L98H | t02m1.1c09e05 |
| B4 | 29/11/2021 | 230 | NS | **16** | 2* | **1** | **16** | TR34/L98H | T02m1.1c09e05 |
| B8 | 11/04/2022 | 475 | NS | **16** | 2* | **0.50** | **8** | TR34/L98H | T02m1.1c09e05 |
| B8 | 11/04/2022 | 477 | NS | **16** | 2* | **0.50** | **16** | TR34/L98H | t02m1.1c09e05 |
| B9 | 17/05/2022 | 541 | NS | **16** | 2* | **2** | **8** | TR34/L98H | t02m1.1c09e05 |
| A10 | 29/11/2021 | 225 | NS | **16** | 2* | **0.50** | **16** | TR34/L98H | t02m1.1c09e05 |
| B4 | 29/11/2021 | 227 | NS | **16** | 2* | **0.50** | **16** | TR34/L98H | T02m1.1c09e05 |
| B9 | 17/05/2022 | 537 | NS | **16** | 2* | **0.50** | **8** | TR34/L98H | t02m1.1c09e05 |
| B9 | 17/05/2022 | 543 | NS | **16** | 2* | **2** | **8** | TR34/L98H | t02m1.1c09e05 |
| B9 | 17/05/2022 | 544 | NS | **16** | 2* | **2** | **8** | TR34/L98H | t02m1.1c09e05 |
| B9 | 17/05/2022 | 545 | NS | **16** | 2* | **2** | **8** | TR34/L98H | t02m1.1c09e05 |
| B9 | 17/05/2022 | 542 | NS | **16** | 2* | **2** | **8** | TR34/L98H | t02m1.1c09e05 |
| B1 | 19/07/2021 | 157 | NS | **16** | **8** | **1** | **4** | TR34/L98H | t02m1.1c09e05 |
| B1 | 19/07/2021 | 153 | NS | **16** | **8** | **2** | **8** | TR34/L98H | t02m1.1c09e05 |
| A15 | 17/05/2022 | 510 | NS | **16** | **8** | **0.50** | **16** | TR34/L98H | t02m1.1c09e05 |
| A15 | 17/05/2022 | 500 | NS | **16** | **4** | **2** | **16** | TR34/L98H | t02m1.1c09e05 |
| B9 | 17/05/2022 | 540 | NS | **16** | 2* | **1** | **4** | TR34/L98H | t02m1.1c09e05 |
| A15 | 17/05/2022 | 501 | NS | **16** | **4** | **1** | **16** | TR34/L98H | t02m1.1c09e05 |
| A15 | 17/05/2022 | 505 | NS | **4** | **4** | **0.50** | **8** | TR34/L98H | t02m1.1c09e05 |
| A15 | 17/05/2022 | 502 | NS | **16** | **4** | **1** | **16** | TR34/L98H | t02m1.1c09e16 |
| A15 | 17/05/2022 | 494 | NS | **16** | **4** | **2** | **8** | TR34/L98H | t02m1.1c09e16 |
| A7 | 19/07/2021 | 114 | NS | **8** | **4** | **1** | **4** | TR34/L98H | t02m1.1c09e22 |
| A12 | 28/02/2022 | 323 | S | ND | | | | | t02m1.2c09e05 |
| B6 | 28/02/2022 | 344 | S | ND | | | | | t02m1.2c09e05 |
| B6 | 28/02/2022 | 351 | S | ND | | | | | t02m1.2c09e05 |
| B6 | 28/02/2022 | 353 | S | ND | | | | | t02m1.2c09e05 |
| A15 | 17/05/2022 | 482 | NS | **16** | 2* | **0.50** | **8** | WT | t03m1.1c05Ae07 |
| A15 | 17/05/2022 | 489 | NS | **16** | 2* | **1** | **4** | WT | t03m1.1c05Ae07 |
| A15 | 17/05/2022 | 495 | NS | **16** | 2* | **1** | **4** | WT | t03m1.1c05Ae07 |
| A15 | 17/05/2022 | 496 | NS | **16** | 2* | **1** | **4** | WT | t03m1.1c05Ae07 |
| A15 | 17/05/2022 | 522 | NS | **4** | 2* | **2** | **4** | WT | t03m1.1c05Ae07 |
| A15 | 17/05/2022 | 524 | NS | **4** | 2* | **2** | **4** | WT | t03m1.1c05Ae07 |
| A15 | 17/05/2022 | 506 | NS | 2* | **4** | 0.25* | **8** | WT | t03m1.1c08Ae08 |
| A15 | 17/05/2022 | 513 | NS | 2* | 2* | 0.125 | **4** | WT | t03m1.1c08Ae08 |
| A12 | 28/02/2022 | 320 | NS | 2* | 0,5 | 0.125 | **4** | WT | t03m1.1c08Ae08 |
| A15 | 17/05/2022 | 504 | NS | 1 | 2* | 0.25* | 2* | WT | t03m1.1c08Ae08 |
| A15 | 17/05/2022 | 523 | NS | **4** | 2* | **2** | **4** | WT | t03m1.1c09e07 |
| B4 | 29/11/2021 | 235 | NS | 2* | 0,5 | 0.25* | **4** | WT | t03m1.1c10e06 |
| B4 | 29/11/2021 | 236 | NS | 2* | 0,5 | **0.50** | **8** | WT | t03m1.1c10e06 |
| A13 | 22/03/2022 | 371 | S | ND | | | | | t03m1.1c10e09 |
| A15 | 17/05/2022 | 498 | S | ND | | | | | t03m1.3c08Ae07 |
| A15 | 17/05/2022 | 518 | S | ND | | | | | t03m1.3c08Ae07 |
| A14 | 11/04/2022 | 456 | S | ND | | | | | t04Am1.1c08Be06 |
| A11 | 18/01/2022 | 242 | S | ND | | | | | t04Am1.2c08Ae15 |
| A11 | 18/01/2022 | 254 | S | ND | | | | | t04Am1.2c08Ae15 |
| A11 | 18/01/2022 | 263 | S | ND | | | | | t04Am1.2c08Ae15 |
| B5 | 18/01/2022 | 273 | S | ND | | | | | t04Am1.2c08Ae15 |
| B5 | 18/01/2022 | 275 | S | ND | | | | | t04Am1.2c08Ae15 |
| A15 | 17/05/2022 | 478 | S | ND | | | | | t04Am1.3c05Ae07 |
| B10 | 13/06/2022 | 572 | NS | **4** | **4** | **1** | **8** | WT | t04Am1.3c08Be07 |
| B4 | 29/11/2021 | 231 | S | ND | | | | | t04Am3.4c05Ae11 |
| A12 | 28/02/2022 | 284 | S | ND | | | | | t04Am3.4c17e11 |
| A14 | 11/04/2022 | 448 | NS | **16** | **4** | **1** | **16** | TR34/L98H | t04Bm1.2c08Ae07 |
| B1 | 19/07/2021 | 163 | S | ND | | | | | t05m1.1c03e13 |
| B8 | 11/04/2022 | 463 | S | ND | | | | | t05m1.3c03e13 |
| B8 | 11/04/2022 | 474 | S | ND | | | | | t05m1.3c03e13 |
| B9 | 17/05/2022 | 532 | S | ND | | | | | t05m1.3c03e13 |
| B3 | 27/09/2021 | 209 | S | ND | | | | | t05m1.3c08Ae16 |
| B1 | 19/07/2021 | 146 | NS | **16** | **8** | **0.50** | **8** | WT | t09m1.1c04e13 |
| B1 | 19/07/2021 | 136 | NS | **8** | **4** | **0.50** | 2* | WT | t09m1.1c04e13 |
| B1 | 19/07/2021 | 142 | NS | 1 | 2* | **1** | **8** | WT | t09m1.1c04e13 |
| B1 | 19/07/2021 | 140 | NS | 1 | **16** | **1** | **4** | WT | t09m1.1c04e13 |
| Strains were classified as susceptible (S) or not susceptible (NS) to at least one of the tested azoles in the screening. Resistance for itraconazole (IZ), voriconazole (VZ), posaconazole (PZ) and isavuconazole (ISV) following EUCAST breakpoints are stated in bold. * ATU. MICs of susceptible strains from the screening were not done (ND) but those that were typed are included in this table. | | | | | | | | | |
